# Digital Filtering of Railway Track Coordinates in Mobile Multi–Receiver GNSS Measurements

**DOI:** 10.3390/s20185018

**Published:** 2020-09-04

**Authors:** Andrzej Wilk, Wladyslaw Koc, Cezary Specht, Slawomir Judek, Krzysztof Karwowski, Piotr Chrostowski, Krzysztof Czaplewski, Pawel S. Dabrowski, Sławomir Grulkowski, Roksana Licow, Jacek Skibicki, Mariusz Specht, Jacek Szmaglinski

**Affiliations:** 1 Faculty of Electrical and Control Engineering, Gdańsk University of Technology, 80–233 Gdańsk, Poland; andrzej.wilk@pg.edu.pl (A.W.); wladyslaw.koc@pg.edu.pl (W.K.); krzysztof.karwowski@pg.edu.pl (K.K.); jacek.skibicki@pg.edu.pl (J.S.); 2 Faculty of Navigation, Gdynia Maritime University, 81-374 Gdynia, Poland; c.specht@wn.am.gdynia.pl (C.S.); k.czaplewski@wn.umg.edu.pl (K.C.); p.dabrowski@wn.am.gdynia.pl (P.S.D.); m.specht@wn.am.gdynia.pl (M.S.); 3 Faculty of Civil and Environmental Engineering, Gdańsk University of Technology, 80-233 Gdańsk, Poland; piotr.chrostowski@pg.edu.pl (P.C.); slawomir.grulkowski@pg.edu.pl (S.G.); roksana.licow@pg.edu.pl (R.L.); jacek.szmaglinski@pg.edu.pl (J.S.)

**Keywords:** track geometry surveying, GNSS mobile measurements, condition monitoring, Savitzky–Golay filter, Whittaker filter, sensor data pre–processing

## Abstract

The article discusses an important issue in connection with the technique of mobile Global Navigation Satellite System (GNSS) measurements of railway track coordinates, which is digital filtering performed to precisely determine railway track axes. For this purpose, a measuring technique is proposed which bases on the use of a measuring platform with a number of appropriately distributed GNSS receivers, where two of them determine the directional base vector of the platform. The receivers used in the research had high measuring frequency in the Real Time Kinematic (RTK) operating mode and enabled correction of the obtained results in post–processing. A key problem discussed in the article is the method for assessing the quality of the measurement results obtained from GNSS receivers, and their preparation for further processing making use of geometrically constrained parameters of the base vector and specialized digital filtering, among other elements, to precisely determining the track axis. The obtained results confirm the applicability of the used method of GNSS signal processing.

## 1. Introduction

In terms of their shaping, railway tracks have a precisely defined geometric layout which determines the maximum acceptable speed of trains running on them. Therefore, attempts have been made to find the best method to describe the geometric layout of the rail track axis, as it is subject to deformations during exploitation. The International Union of Railways (UIC) reported, at the end of 2017, that there are about 1140 thousand km of railway tracks in operation, annual passenger transport reaches as much as nearly 3940 billion passenger–km, while cargo transport stands at 10665 billion tonne–kilometres. Consequently, each failure of railway infrastructure leads to substantial transport disturbances and generates costs. For this reason, periodic inspection of the technical condition of a railway transport system is essential for maintaining its high reliability and safety. One method for monitoring the shape of the rail track axis consists of performing periodic control measurements of geodetic coordinates of the track, which are then used to determine its real axis and compare it with the design. The shape of the axis cannot take the form characterised by variable curvature over a relatively long distance [1,2,3,4]. This issue gains ever more importance when taking into account details of the technique for cataloguing measurements of a railway track [5,6,7,8,9,10,11,12] and methods to determine the shape of its axis [3,13,14]. The coordinate measuring is usually done using a surveying instrument installed at some height above the track axis. This is also the case in the recently introduced and commonly used method based on a mobile total station, which enables continuous measurement of coordinates. The measurements make use of a special railway track geodetic reference frame. Computations are made in the Cartesian coordinate system, PL–2000 in Poland (Gauss–Krüger projection for World Geodetic System–WGS 84), therefore, correcting the recorded coordinates of the existing track axis requires determining railway grid directions in the horizontal plane at each measuring point, which can be difficult for points situated on an arc. This approach is both time–consuming and disruptive to normal operations on the railway line. Furthermore, its results can depend on a subjective assessment by the personnel performing the measuring.

The increasing accuracy of GNSS measurements provides new opportunities for developing effective inspection methods and designing track axis adjustment projects. Numerous tests have proved that for mobile GNSS receivers, it is possible to achieve a positioning accuracy of about 0.5 cm. The main advantages of the measuring wagon–platform based multi–sensor GNSS measurement method [15,16,17,18] are its mobility, high measuring rate, high positioning accuracy, and the lack of any need to perform relatively complex transformations to assess results as well as the possibility to automatize the measurements. Compared to tacheometric methods, the GNSS method does not require the constructing and maintaining of a specialized surveying grid. Basic difficulties in its application result from the high technological advancement of measuring instruments (availability, service) and the need to operate on large sets of measured data. The developed methods of mobile multi–receiver GNSS measurements outperform the remaining stationary and quasi mobile techniques (i.e., those with a measuring trolley guided manually by the operator) [19,20]). This results from the fact that in this method, the time needed for precise geodetic coupling to physical infrastructure situated along the track is omitted, and the synchronized measuring signals are recorded with a relatively high frequency. In many countries, GNSS techniques are introduced to support determining of track coordinates; however, their use for accurate (in the global sense) mapping of track axis, especially in areas with varying access to satellites, is still a challenging issue [21,22,23,24,25,26,27,28,29,30,31,32,33,34]. That is why the research project InnoSatTrack included an attempt to build a platform for multi–sensor track geometry measurement [35].

In the article, the authors report the results of the comparative analysis of data recorded by a multi–receiver GNSS mobile platform during measurements performed on a selected railway line section. A new task was, therefore, formulated, using of geometrically constrained GNSS receivers forming the base vector of the measuring vehicle, referring to its modulus and direction, for evaluating the quality of the measurement. The proposed multi GNSS structure of the measuring system provides opportunities for further consideration of the vector perpendicular to the base vector as well as considering a single measurement as a quasi–repeated measurement or making use of the so–called parametric method for observational adjustment. Attention has also been paid to the presence of incorrect measurement results generated by disturbances in the GNSS signal recording and by relatively small, compared to an urban canyon for instance, infrastructure obstacles, such as railway and road viaducts. An algorithm is proposed for detecting incorrect data with the use of the Savitzky–Golay filter [36]. All this information was used in the algorithm for filtering the disturbed GNSS coordinates. For this purpose, the Whittaker filter was used, which belongs to the group of discrete penalized least square filters and is resistant to relatively long intervals of data absence in measuring data sequences [37,38]. It is noteworthy that this is the first application of this type of GNSS data filtering to precisely determine the railway track axis coordinates.

## 2. Mobile Multi GNSS Measuring Platform

GNSS measurements can generally be divided into either static or kinematic [18]. Static measurements include a large amount of excess information. In the offline process, this data is corrected based on the information from the reference stations, thus ensuring the highest accuracy of measurements; however, this method cannot be applied in this type mobile measurements. For the GNSS receiver in motion, the results of measurements refer to the current position in time, and this position is determined based on observations from a number of surveying epochs, which does not ensure as high a level of accuracy as that in stationary measurements. The precise position measurement of a receiver in motion can be done in RTK mode. However, in this case, direct communication with the reference station or a system of reference stations in Real Time Network (RTN) mode is required [16]. Since this communication is not always fully provided in the railway line areas, a kinematic method should be used in post–processing, which means that the position measurements of the receiver in motion should include correcting calculations performed after the measurement has been completed.

Mobile measurements require GNSS receivers with the option of precise kinematic measurement and high–frequency data recording, 20 Hz for instance. This particularly refers to measurements performed at different receiver speeds and/or in different environmental and terrain conditions. When a single GNSS receiver is used, possible measuring errors may exceed acceptable levels, which decreases the final accuracy of the railway track axis coordinates evaluation. Applying several receivers increases the density of measurements as well as enabling the evaluation of their accuracy and eliminating incorrect results. Moreover, it enables obtaining a number of additional parameters describing the railway track geometry [16,17,18,39,40,41,42].

The track axis coordinate measurement should be performed using the instrumentation situated in the track cross section axis, and the measuring sensor should be placed on the plane connecting the heads of the two rails forming the track. Because of structural restrictions, among other factors, this arrangement is not possible in mobile GNSS measurements. Instead, the GNSS receiver is installed at some height over this plane, resulting from the overall dimensions of the measuring platform, the dimensions of the system fixing the receiver to its frame, and the height of the receiver itself. As a consequence, all longitudinal and/or lateral track inclinations, in particular those characteristic of horizontal track arcs with constant or varying curvatures, cause that the real horizontal receivers’ position differs from the reference position. In this situation, the track axis coordinates measured by the GNSS receiver are subject to an error which should be eliminated. Correction of the recorded coordinates is possible if other sensors, measuring longitudinal and lateral inclination angles of the measuring platform frame, are installed close to the GNSS receiver. Mobile measurements make use of various specially designed or standard versions of wagons, motor cars, and locomotives. The higher the receiver is installed relative to the track plane, the greater the effect on the track axis evaluation accuracy. Increasing this accuracy requires relevant post–processing with combined use of the results obtained from both GNSS receivers and inclinometers.

**Figure 1:**
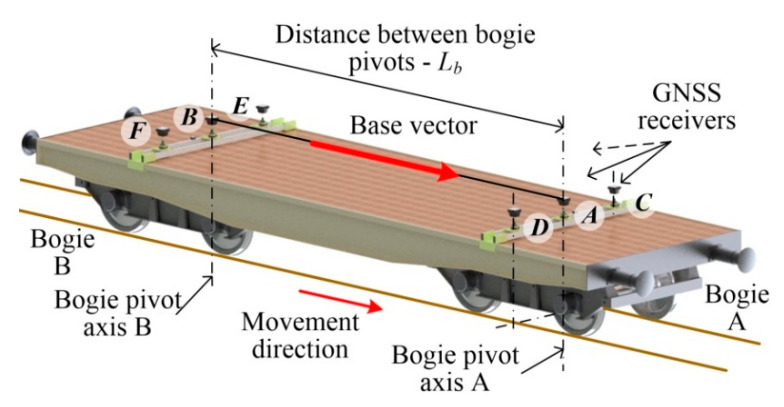
Mobile measuring platform with receivers *A*, *B*, …, *F* and marked base vector $\vec{AB}$.

Figure 1 shows a concept for a mobile measuring platform with six GNSS receivers distributed on a typical flatcar. Two receivers *A* and *B*, which are of particular importance in the presented method, are situated above the bogie pivot pins, along their vertical axes. They form the so–called fixed base of length *L_b_*. Taking into account the movement direction of the platform, the base vector marked in the figure was obtained. The coordinates of GNSS receivers composing the base vector $\vec{AB}$ can be used for evaluating the correctness of track axis location measurements in the global coordinate system.

**Figure 2:**
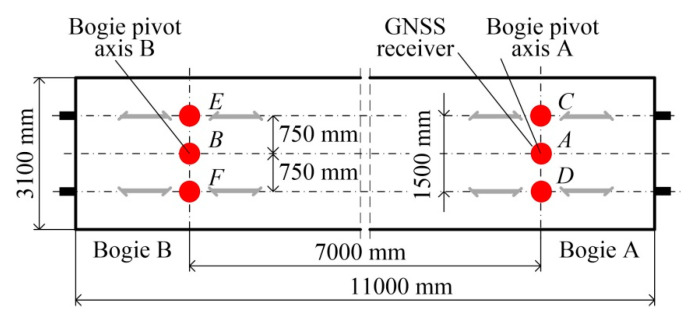
Layout of GNSS receivers *A*, *B*, …, *F* on measuring platform.

This concept was implemented using a standard flatcar (type 401Z, Figure 1). The main parameters of this platform are: mass—19.9 × 10^3^ kg, loading deck height above rail head—1292 mm, frame length—11000 mm, frame width—3100 mm, distance between boogie pivot pins—7000 ± 10 mm, transverse tolerance in pivot axis distribution +2/−3 mm, boogie axis spacing—2000 mm, arc passing—90 m, maximum speed—100 km/h. The GNSS receivers’ layout on the platform and the distances between them are schematically shown in Figure 2. The method of single direct measurement was used to measure the wagon’s symmetry axes, i.e., the pivot pin axes, in such a way that the tolerance of the layout of GNSS receivers was within ± 1 mm.

**Figure 3:**
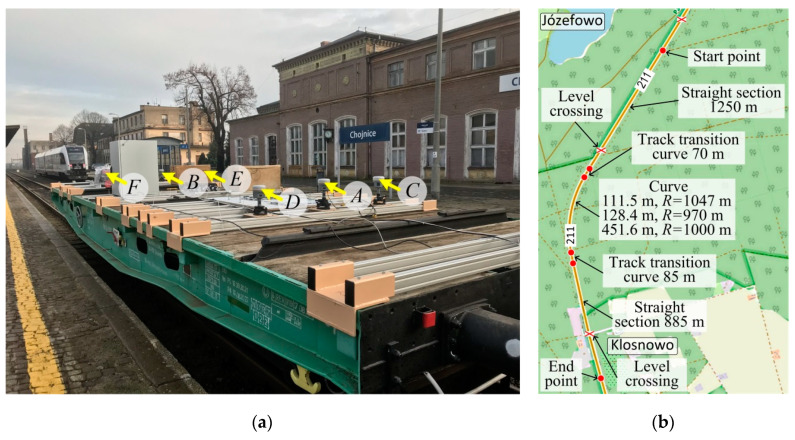
Test stand: (**a**) view of mobile measuring platform with receivers *A*, *B*, …, *F* installed on 401Z flatcar; (**b**) general map of part of railway line No. 211 between Chojnice and Brusy stations (https://www.openrailwaymap.org).

Figure 3a shows a view of the measuring platform with GNSS receivers and the remaining measuring and DAQ (data acquisition equipment). The tribraches and GNSS receivers were mounted on aluminium profiles transversely fixed to the flatcar. The measuring train consisted of a 401Z flatcar, on which the measuring and DAQ equipment was installed, an additional separating wagon, and a DH–350 motor car. The additional wagon was used to increase the distance between the platform and the motor car cab, thus reducing the risk that the GNSS receivers are disturbed by the cab during the measurement.

To allow the proper interpretation of the results of GNSS measurements, they are presented, for both stationary and mobile mode, in the Cartesian coordinate system *Y*, *X* on *Y*(*t_i_*), and *X*(*t_i_*) plots for both curved and straight railway line sections. The results are compared between different GNSS receivers, in particular between receivers *A* and *B* defining the fixed base of the measuring platform.

## 3. Results of Multi GNSS Measurements of Railway Track

### 3.1. Stationary Measurements

**Figure 4:**
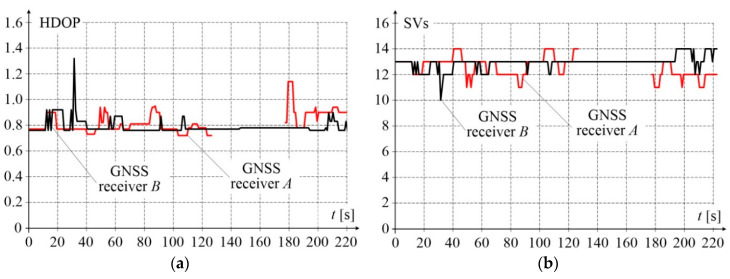
Measurement quality indicators of receivers *A* and *B*: (**a**) HDOP characterising the position accuracy in 2D space; (**b**) number of space vehicles.

The presented results of measurements were recorded during the experimental ride performed in December 2019 on the test stand shown in Figure 3b. The measurements were done using new–generation GNSS receivers and the network of reference stations RTK/RTN operating in Poland. The applied GNSS receivers, model R10 made by Trimble, were fixed on the platform as shown in Figure 2. During a relatively short platform stop of 3.7 min, before starting mobile measurements, each receiver collected 4440 records. The receivers acquire raw data with 20 Hz frequency, four of them operated simultaneously in RTK 1 Hz mode, and one in 20 Hz mode. Selected quality indicators describing the effect of the almanac on position evaluation are shown in Figure 4. The presented data were collected at one second intervals during the entire static condition time. Both the horizontal dilution of precision (HDOP), which did not exceed a value of 1.3, and the number of space vehicles (SVs), which was not less than 10, correspond to the excellent rating. At this level of parameters, positional measurements are considered accurate enough to meet the majority of railway applications.

**Figure 5:**
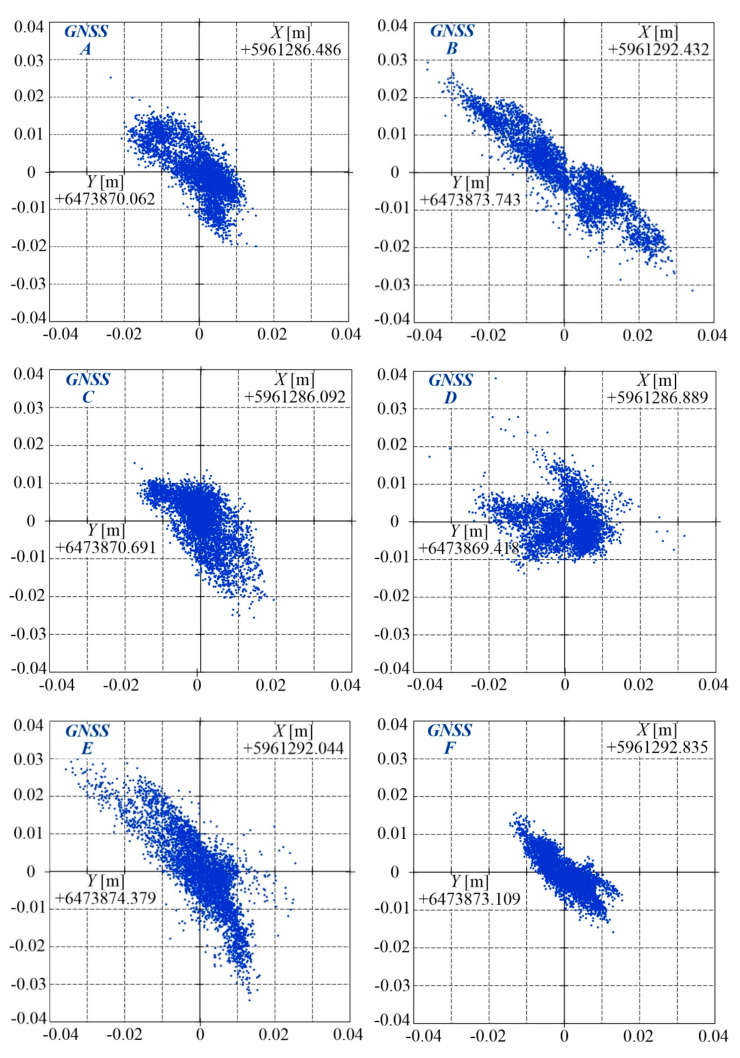
Results of position measurements for receivers *A*, *B*, …, *F* on static condition.

For the results collected in the above way, RTN corrections were introduced in post–processing. The corrected results of stationary measurements are shown in Figure 5, in the Cartesian coordinate system WGS 84 (in Poland PL–2000), for each of receivers *A*, *B*, …, *F*. Each position was determined in coordinates *Y*, *X*, where the *Y* values increases from West to East, while the *X* values increases from South to North.

Based on the values acquired by receivers *A*, *B*, …, *F*, their arithmetic means were calculated and taken as position coordinates. The standard deviations of these positions were also calculated. The obtained results are collated in Table 1. For the presented measurements, the maximum standard deviation was slightly above 1 cm.

The results obtained from the GNSS receivers were used for calculating the distances between antennae and the slopes of lines passing through the measuring points. The lines to be used for determining the railway track axes are those passing through points *A*–*B*, *C*–*E*, and *D*–*F* as well as the lines determined from measurements for three points: *C*–*A*–*D* and *E*–*B*–*F* (Figure 2). Table 2 compares characteristic distances between receivers which were determined from GNSS measurements with those measured directly using a total station and class I measuring tape. The relative error of fixed base vector $\vec{AB}$ length is less than 0.1%.

After expressing in angles the line slopes between the abscissa and a given line passing through the central points of the receivers, the mean value was calculated for the three angles for lines *A*–*B*, *C*–*E*, and *D*–*F*, which was equal to 58.208° with maximum deviation of 0.04°, and another mean value for lines *C*–*A*–*D* and *E*–*B*–*F* (perpendicular to lines *A*–*B*, *C*–*E*, *D*–*F*), which was equal to 31.983° with maximum deviation of 0.07°. 

These results reveal that the slope for the line directed along the measuring platform was determined more precisely than for the line in the normal direction. The sum of these two angles, which should theoretically equal 90°, differs by 0.2° from this value. Here, and of high importance, is the layout tolerance of the receivers–changing the receiver’s position by ±1 mm changes the inclination angle by 0.02° for lines directed along the platform and by as much as 0.08° for lines in the normal direction.

The coordinates of the receivers composing the base vector were used for evaluating the correctness of track axis position measurements in the global coordinate system. After comparing the results shown in Figure 5 with the dimensions of the antenna layout on the platform, measured by independent methods, a conclusion can be made that the quality of the results in stationary measurements is good and the maximum absolute errors are close to 1 cm.

### 3.2. Mobile Measurements on Straight Track Section

**Figure 6:**
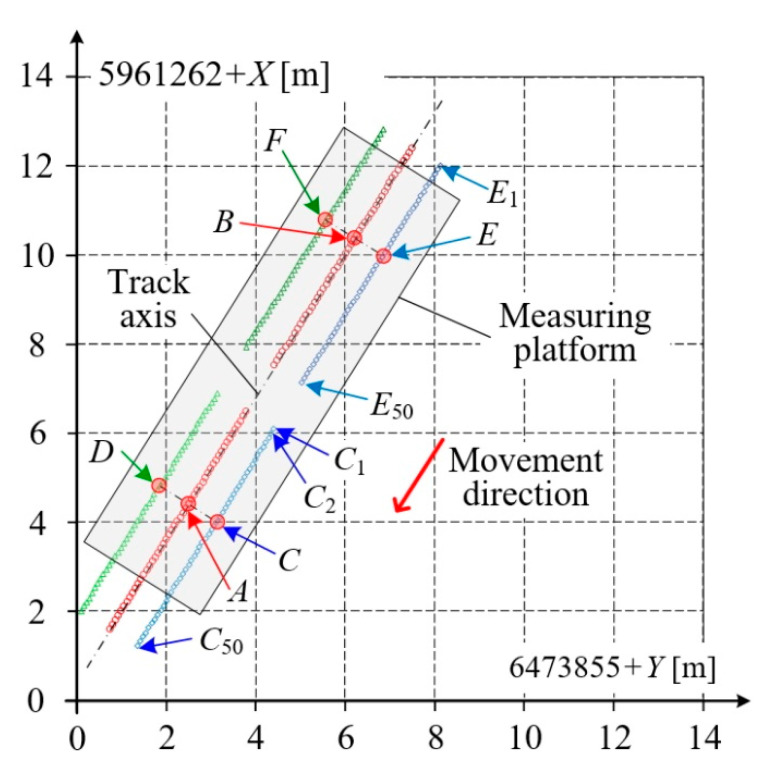
Results acquired on the straight horizontal track.

The GNSS receivers used in the research acquire the measurements synchronically with a frequency of 20 Hz. During the measurement, the receiver’s coordinates *Y*(*t_i_*), *X*(*t_i_*) are acquired with time interval *t_i+1_*_−_ *t_i_* = 50 ms for each of receivers *A*, *B*, … *F*. Graphic interpretation of the results obtained during 2.5 s of acquisition on the straight horizontal track section are shown in Figure 6. This shows 50 samples for each receiver. For instance, receiver *C* obtained: *C_1_*, *C_2_*, …, *C_50_*, where *C*(*t_i_*) = *C_i_*. In the described situation, the speed of the measuring platform was relatively low and the trace of the measurements recorded by receivers *A*, *C*, *D* does not coincide with that of receivers *B*, *E*, *F*. Thus, the interpretation of the results of mobile measurements differs from that of the stationary measurements performed on the static condition. A single measurement result is obtained for each position of the measuring platform. A higher density of measurements can be achieved by using a larger number of receivers, or receivers operating with higher frequency, 100 Hz for instance.

In static conditions, the results are most frequently averaged, while for mobile measurements, results which are evidently incorrect are omitted. The quality evaluation criterion for the obtained measurement results can be formulated based on samples acquired by several GNSS receivers, in particular, the most reduced system of two receivers *A*, *B* composing the base vector $\vec{AB}$ of the measuring platform (Figure 1).

**Figure 7:**
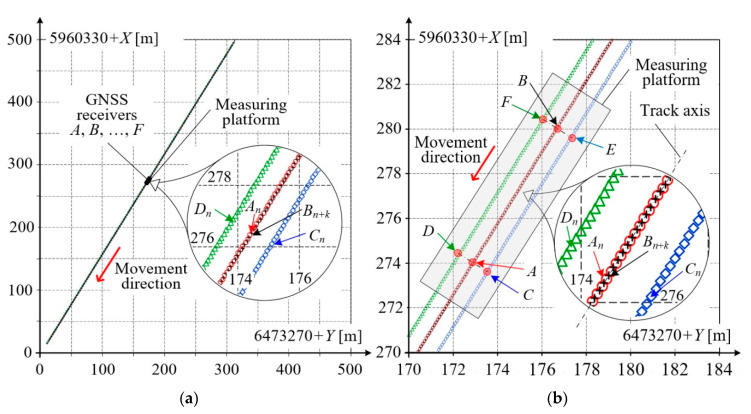
Results acquired by multi GNSS platform: (**a**) for longer section on straight railway track; (**b**) in expanded scale for receivers *A*, *B*, *C* and *D*.

Figure 7 presents selected results of measurements for receivers *A*, *B* form the base vector of the measuring platform, and – for better clarity – receivers *C*, *D*. The platform position and dimensions are also sketched symbolically onto the figure. These results are partially repeated in the expanded scale of several meters in Figure 7b, i.e., the result *A*(*t_n_*) = *A_n_* for time *t_n_*. Since the receivers *A*, *B* are geometrically constrained to compose the base vector, they are placed in the symmetry axis of the platform and, as a consequence, after time *t_k_* the receiver *B* will acquire a sample in the vicinity of sample *A_n_*. A similar situation occurs for receivers *C*–*E* and *D*–*F*. This result will be marked *B_n+k_*, where *k* depends on the platform’s speed. Since the length of the base vector is *L_b_* = 7000 mm and the speed of platform was *v* = 2.84 m/s, then in this case *k* = 50, and *t_k_* = 2.5 s.

**Figure 8:**
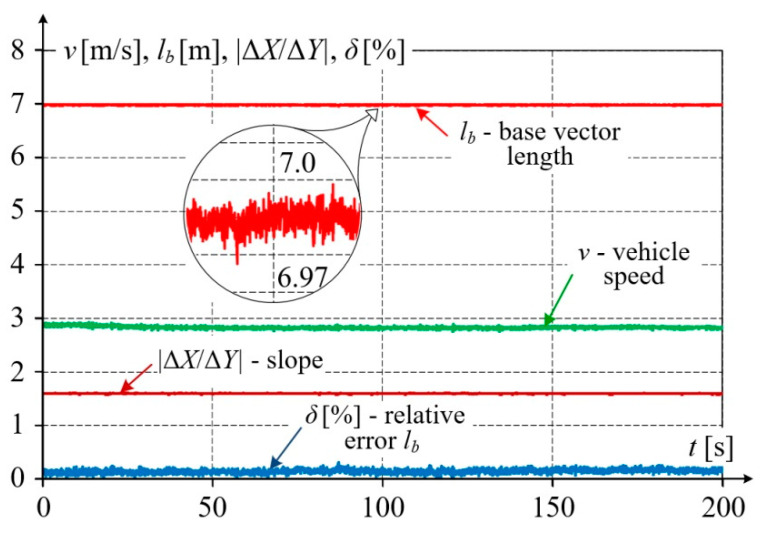
Waveforms of qualitative parameters of measurements on a straight railway track section—determined for base vector $\vec{AB}$.

During mobile GNSS measurements, both the environmental and terrain conditions vary, which affects the quality of the obtained results. Figure 8 shows characteristic base vector parameters as elements of a qualitative evaluation of the measurement made by receivers *A*, *B* form this vector. 

The distance between these receivers measured directly was *L_b_* = 7000 mm, while the distance calculated based on *Y*, *X* coordinates (marked *l_b_*) is close to this value. The relative error of the base vector length did not exceed 0.24%. The slope of this vector, marked Δ*X*/Δ*Y* and calculated as the difference quotient (*X_n_*_+1_ − *X_n_*)/(*Y_n_*_+1_ − *Y_n_*), varied slightly from 1.594 to 1.608, i.e., was nearly constant along this straight section of railway track. The vehicle speed *v* shown in Figure 8 was obtained from components *v_Y_*, *v_X_*, calculated as difference quotients of vehicle position changes to sampling times. Visible small speed variations result from the limited accuracy of measurements and numerical rounding in a relatively short measuring cycle of 50 ms. The vehicle speed was practically constant and approximately equal to *v* = 2.83 m/s.

During the post–processing of mobile measurement results, the sporadic appearance of samples with large deviations of coordinate values from those recorded by other receivers was observed. These errors, seen in sequences from a few to several dozen samples taken from different GNSS receivers, were not caused by environmental disturbances.

**Figure 9:**
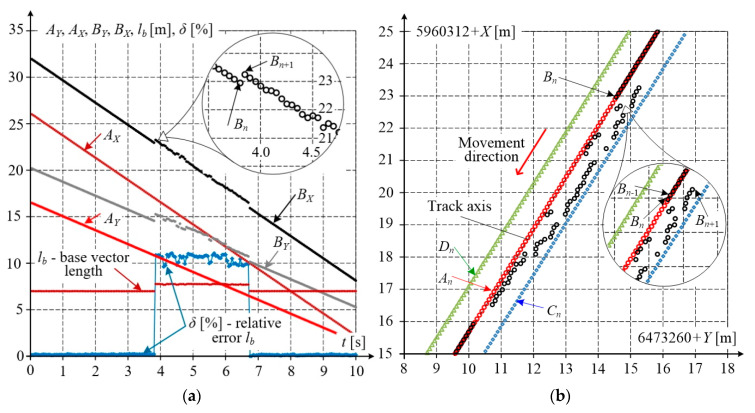
Example of incorrect dataset: (**a**) waveforms of correct and incorrect results acquired by receivers *A* and *B*, complemented by the calculated base length *l_b_* and its relative error *δ*; (**b**) correct results acquired by receivers *A*, *C* and *D*, and incorrect results acquired by receiver *B*.

Figure 9a shows the coordinate changes for receivers *A*, *B*. The correct waveform is observed for coordinates *A_i_* while for coordinates *B_i_*, it reveals some disturbances due to incorrect measurements. These incorrect samples can be easily detected in the set of acquired data by comparing the calculated (*l_b_*) and directly measured (*L_b_*) base vector lengths. Since the platform motion on the track is well defined and cannot change rapidly, calculating difference quotients of coordinates will enable identification of the receiver with incorrect measuring data. The base length *l_b_* calculated from GNSS coordinates and compared with length *L_b_* is one of the quality evaluation criteria for both measurements alone as well as their further processing and filtering. Figure 9a shows a noticeable change of the base length *l_b_*, determined with relative error *δ* of more than 10%. Using the expanded scale, discrete measuring points are shown for receiver *B* and coordinate *B_X_* – the observed step change is of the order of 0.5 m.

Figure 9b shows the same part of the dataset in the Cartesian coordinate system *Y*, *X*. Correct measurement results were visibly acquired by receivers *A*, *C,* and *D*, (to preserve the clarity of the picture, the results from receivers *E* and *F* are omitted). Along the horizontal straight track section, the track axis can be easily determined, and this axis should coincide with the results acquired by receiver *B*. Correct results (black circles) can be seen in the upper and lower parts of the picture, while the middle part reveals large deviations. This effect is particularly noticeable when comparing correct sample *B*(*t* = *t_n_*) = *B_n_* with incorrect sample *B_n_*_+1_, and a further 50 samples collected with the sampling time of 50 ms. This sequence of coordinates reveals large measuring errors. The measuring platform moved at a relatively low speed, *v* = 10 km/h, and covered a distance of about 7.08 m during the recorded time of 2.55 s.

**Figure 10:**
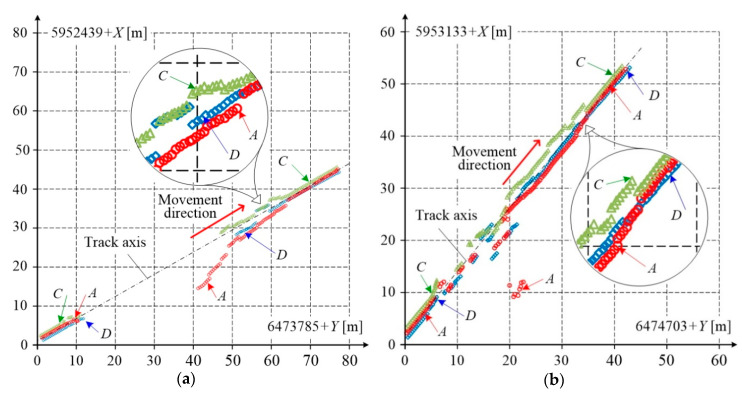
Results of GNSS signal loss when the measuring platform passed: (**a**) under road viaduct; (**b**) under lattice truss railway viaduct.

The satellite signal can be lost when the platform passes some environmental disturbances along the way. In this case, the GNSS receiver either does not generate results at all, or the results are recorded irregularly. Samples showing breaks in the receivers’ operation, after interrupting of the measuring platform by road and railway viaduct, are shown in Figure 10. When the platform passed under the road viaduct, the signal was completely lost by all receivers, while when passing under a lattice truss railway viaduct, the signal loss was temporary and its intensity varied for each receiver.

Figure 10 shows the results of measurements acquired by three GNSS receivers: *A*, *C*, and *D*. As the measuring platform passes under the road viaduct, the measurement results reveal a noticeable break. At the platform speed *v* = 30 km/h, this break was approximately equal to 10 s and the measurement results in this situation are burdened with extremely large errors. Furthermore the time after which correct results can be acquired once the obstacle has been passed is relatively long.

In the above situation, to more precisely determine the railway track axis, the results which are missing due to satellite signal loss should be complemented using other measuring techniques, for instance, an inertial measurement unit (IMU) with a Kalman filter algorithm, frequently used for this purpose e.g. [43]. When the time of signal loss is relatively short, incorrect samples can be identified using the measuring platform base vector, and then corrected using relevant error reduction algorithms.

### 3.3. Mobile Measurements on Railway Track Arc Section

**Figure 11:**
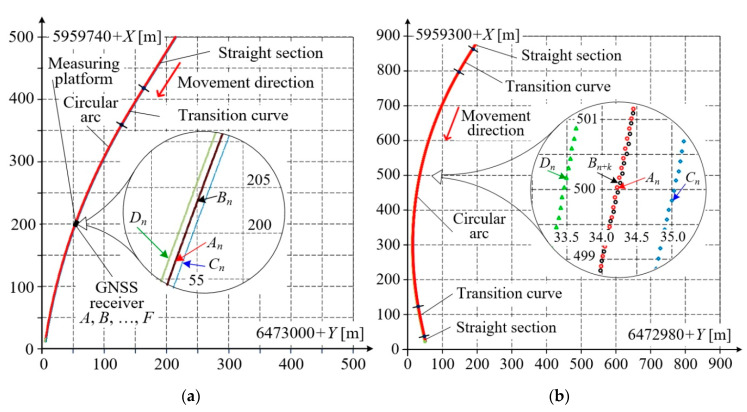
Results acquired on a geometrically complex railway track segment: (**a**) straight section changing to circular arc; (**b**) section consisting of transition curves and circular arc.

Figure 11 shows the results of measurements performed on a geometrically complex railway track segment consisting of a straight section and a circular arc linked together by transition curves. Figure 11a refers to the case when the measuring platform moves along the straight section and the transition curve, while Figure 11b covers a longer track section of nearly 1 km in length, with the transition curves and the circular arc. As in previous figures, only the results acquired by receivers *A*, *B* and *C*, *D* are shown. The obtained results were post–processed to determine the base length *l_b_* and slope Δ*X*/Δ*Y* in an way identical manner to that applied in Figure 8.

**Figure 12:**
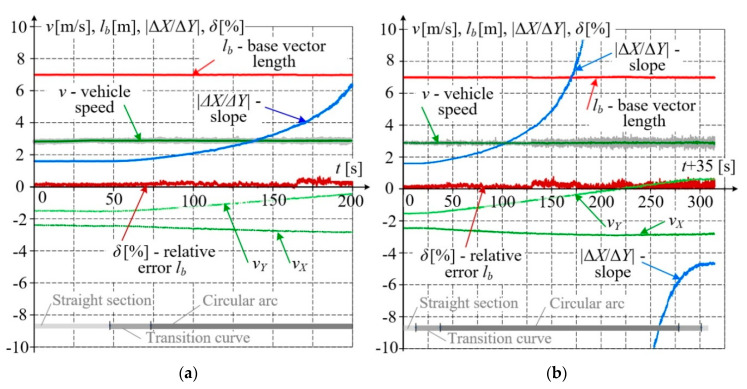
Waveforms of qualitative parameters of multi GNSS measurements: (**a**) straight section of track changing to circular arc; (**b**) section consisting of transition curves and circular arc.

Based on the data presented in Figure 11a,b, the waveforms of selected quality parameters were calculated and shown in Figure 12a,b. The calculated fixed base length is practically constant (the maximum relative error does not exceed *δ*_max_ = 0.7%). The slope changes smoothly from a constant value, characteristic for the straight section, to higher values on the transition curve and circular arc. These values can be extremely high in the areas where the base vector direction coincides with the meridian direction, and in those cases, they should be limited in the numerical procedure. Precise evaluation of such a situation would require transforming the measured coordinates to another global system, rotated about a given angle. It is noteworthy that even when the simplified procedure making use of formulae with difference quotients was applied, no disturbances were observed in slope calculations.

Figure 12 additionally shows velocity vector components *v_Y_*, *v_X_*, and its absolute value *v*. The velocity components were calculated as difference quotients of coordinates. A simple filtration method based on calculating a moving average from 10 measuring samples was applied. The absolute velocity value *v* is presented as the moving average against the non–filtered values. The average speed for the entire test ride was 2.89 m/s.

## 4. Digital Filtering of Railway Track Coordinates

The data obtained from GNSS receivers should be checked with respect to their quality. For this purpose, it is proposed to use the measuring platform base vector and the Savitzky–Golay filter [36] to detect incorrect measurement results. In a further step of the analysis, a smoothing–interpolating Whittaker algorithm is used together with the weight coefficient *P* indicating incorrect data [37]. These filters are well described in the literature and widely used [44,45]. Numerous software packages such as MATLAB or LabVIEW, make it possible to filter datasets based on smoothing algorithms. However, their use for determining railway track coordinates is being proposed for the first time.

The main advantage of the Savitzky–Golay filter (SGF) is that it does not introduce delay. The operation of the SGF results directly from the Stone–Weierstrass theorem. Its essence can be expressed as local polynomial regression (LPR), reached in the convolution process via approximating successive sequences of adjacent data points by low–degree polynomials. This method consists of selecting a symmetric window with respect to the analysed data point and attributing the value of the polynomial function to this point at a central window index. This process is repeated for all points, thus obtaining a smoothed signal and its differences (playing the same role as derivatives for continuous functions). The filter requires parametrization, i.e., assuming the degree *n* of the approximating polynomial and the window dimension *m*. Automatic parametrization of the filter is possible [38].

To detect incorrect measurement results, a difference method similar to those used for filtering continuous signals has been used. The motion of the measuring platform is well defined and cannot change rapidly, therefore, the measurement results are coupled together and carry the same correlated signal, which justifies the use of SGF. The assumed polynomial degree was *n* = 2 and window width *m* = 11. The detection index *P*, defining the measurement correctness of each data point *p_i_*, *i* = 1, 2, 3, …, was assumed as equal to 0—for incorrect data and 1—for correct data. This index was calculated via differentiating, i.e., approximating the second time derivatives of components ∂^2^*Y*/∂*t*^2^ and ∂^2^*X*/∂*t*^2^ with SGF. Then, the values differentiated in the above way underwent thresholding and normalization. This procedure emphasizes the presence of data points with high rates of change, which are considered disturbances in the measuring process and should be eliminated.

In the second stage of data post–processing, the Whittaker algorithm was used [37]. Basic advantages of this algorithm include:

- correct operation even when relatively large parts of values are missing, by introducing the weight coefficient 0 or 1,
- single–parameter control of exit signal smoothness,
- possibility of cross–validation.

The mathematical foundations of the Whittaker algorithm are as follows [37] taking the data set *ξ_i_* of length *N*. The distances, or time intervals, between the data points are equal. A smooth series *σ_λ_* should be found to match *ξ_i_*. To solve this problem, a compromise between two contradictory goals, which are fidelity and roughness of the matching series, should be found. The smoother the series *σ_λ_* the more it will differ from *ξ_i_*.

The level of fidelity of series *σ_λ_* to the data set can be measured as the sum of difference squares:

(1) $$S=\sum_{i=1}^{N}{\left(\xi_{i}-\sigma_{\lambda }\right)}^{2}$$

The roughness of *ξ_i_*, in turn, can be expressed by *N*-order differences. The sum of the difference squares gives a simple and effective measure of roughness of *ξ_i_*:

(2) $$R=\sum_{i=1}^{N}{\left(\Delta^{2}\xi_{i}\right)}^{2}$$

Combining these two goals gives the sum *Q* = *S* + *λR*, where parameter *λ* can be adjusted so as to obtain the right balance between fidelity and roughness. An objective choice of *λ* can be made using, for instance, cross–validation [37].

The essence of the penalized least square method is finding the values of *ξ_i_* which will minimize *Q*. Making use of matrix calculus, *Q* can be expressed as:

(3) $$Q={\left|\xi -\sigma_{\lambda }\right|}^{2}+\lambda {\left|D\xi \right|}^{2},$$

where the symbol |•|^2^ means the quadratic norm of an arbitrary vector, i.e. the sum of squares of its components, and **D** is the matrix such that **Dz** = Δ**z**. Making use of matrix calculus, the relations for the vector of partial derivatives can be found:

(4) $$\frac{\partial Q}{\partial \xi^{T}}=-2\left(\xi -\sigma_{\lambda }\right)+2\lambda D^{T}D\sigma_{\lambda }$$

Making (4) equal to zero, a system of linear equations is obtained:

(5) $$\left(I+\lambda D^{T}D\right)\sigma_{\lambda }=\xi ,$$

where **I** is the unit matrix.

When significant fragments of value are missing in the input signal, the algorithm can be modified to operate effectively in this situation as well. Using the concept of detection index *P*, a formal record of weight vector **P** with components *p_i_* and values 0 or 1, respectively, for missing and correct data is introduced. The resulting fidelity measure is then formulated as:

(6) $$S=\sum_{i=1}^{N}p_{i}{\left(\xi_{i}-\sigma_{\lambda }\right)}^{2}={\left(\xi_{i}-\sigma_{\lambda }\right)}^{T}P\left(\xi_{i}-\sigma_{\lambda }\right),$$

where **P** is the diagonal matrix with values *p_i_* on the diagonal. As a result, the equation system (6) takes the form:

(7) $$\left(P+\lambda D^{T}D\right)\xi_{\lambda }=P\xi$$

In the analysed case, the input data *ξ _i_* to the Whittaker algorithm are the measured coordinates *Y*(*t_i_*) = *Y_i_*, *X*(*t_i_*) = *X_i_*, for *i* = 1, 2, 3,…, where *t_i+1_* − *t_i_* = *T_s_* is the constant sampling time, and the weight vector **P** with data correctness indices *p_i_* is obtained using the Savitzky–Golay filter. The parameter *λ* was assumed to be equal to 1000, based on the performed numerical experiments.

**Figure 13:**
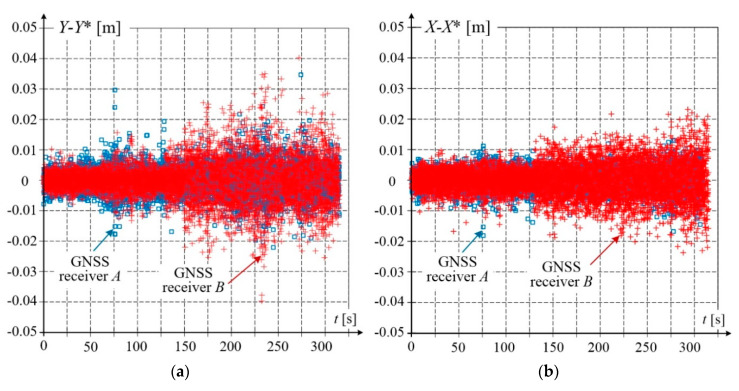
Results of filtration based on Savitzky–Golay and Whittaker algorithms calculated as differences of coordinates before and after filtration: (**a**) coordinate *Y*; (**b**) coordinate *X*.

Qualitative analysis was performed for the measurement results shown in Figure 11b. For receivers *A*, *B*, no incorrect data was acquired, this was also confirmed by the base vector parameters. The *Y*, *X* coordinates of receivers *A*, *B* were filtered using the Whittaker algorithm with parameter *λ* = 1000 to obtain the smoothed values Y*, and X*. Figure 13 shows the differences between the coordinates before and after filtration, i.e., *Y*(*t_i_*) − *Y*(*t_i_*)*, and *X*(*t_i_*) − *X*(*t_i_*)*. The presented results reveal that the filtration smooths the majority of data to within 1 cm.

An important parameter is the measuring vehicle’s speed *v*(*t*), which can be calculated from the vehicle’s position derivatives, in this case: from coordinate differences. The differentiation calculations were performed for SGF windows with 5, 7, 9 and 11 points. For this type of data, with small amplitude changes and small sampling time, the results were close to each other and had slightly smaller amplitude for wider windows.

When analysing the longer section of track from Figure 11b, the applied incorrect data detection algorithm sporadically detected incorrect data for receiver *B*, as sequences of some points in length. Depending on the selected window width *m*, only part of the detected incorrect data points coincided with each other.

**Figure 14:**
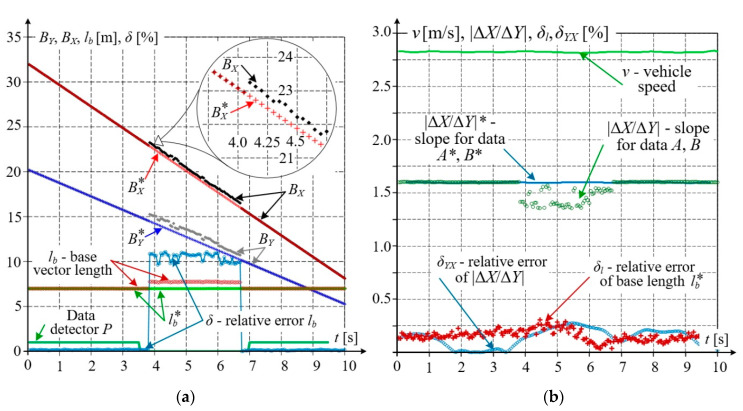
Results of filtering based on Savitzky–Golay and Whittaker algorithms: (**a**) waveform of data detector, correction of receiver *B* results to B*, base vector length and relative error; (**b**) speed, base vector slope, and relative error.

The operation of filters on the data with concentrated incorrect values was checked for the data presented in Figure 9a. The analysis was performed for a selected short dataset consisting of 200 samples with 57 incorrect values. The results of filtration are shown in Figure 14. Figure 14a presents the calculated base vector length *l_b_* and the data detector *P*, which indicates a slightly wider interval of incorrect data due to a relatively large width of the applied window (*m* = 11). The relative error *δ* of length *L_b_* in the area of incorrect data is approximately equal to 10%.

After eliminating incorrect data and processing the remaining data with the Whittaker algorithm, the corrected values B_Y_*, B_X_* were obtained. They are shown in Figure 14a while Figure 14b, being a complement to that data, presents, among other parameters, the base slope related to the reference value for receiver *A* and the value A* obtained from digital filtration. This slope was also determined for non–filtered coordinates from receiver *B* and processed data B*. For the straight railway section, this slope should be constant. The relative error in slope estimation exceeded 10% in some cases.

Figure 14b shows the relative errors of the base vector related to its length and slope. The relative length error *δ_l_* was calculated from the reference data of receiver *A* and the data of receiver *B* after filtration, i.e., B*. The relative slope error *δ_YX_* of this vector was also calculated from data *A*, B*. Higher values of these errors were observed in the incorrect data range. The maximum errors were equal to: *δ_lmax_* = 0.31%, *δ_YX_* = 0.25%.The analysed straight railway track section can be described by the linear equation in the slope–intercept form, which can be obtained using the least square method for coordinate errors of receivers *A*, *B,* and their values after filtration A*, B*, respectively. For receiver *A*, no incorrect data was observed, and the straight line obtained from data *A* or A* can be considered the reference for the data acquired for receiver *B* and their corrected values B*. The difference in values between the reference equation and that obtained from the data for receiver B varies from 0.2 to 0.27 m, while for the equation obtained based on corrected values B*, it is practically constant and equal to 0.01 m. Additionally, Figure 14b shows the vehicle speed *v*, which was obtained from differentiating coordinates after their correction with a 5–point window.

**Figure 15:**
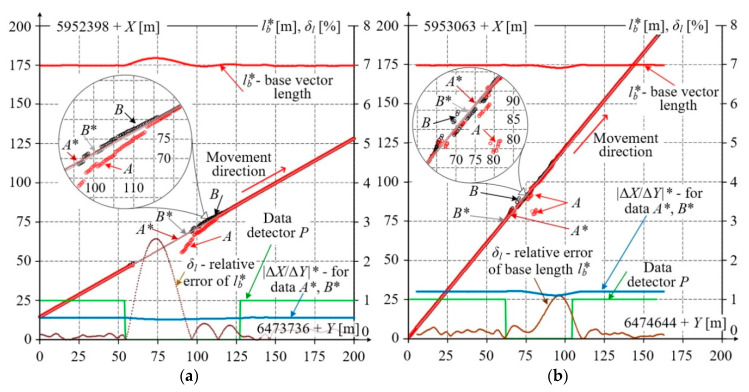
Results of measurements and their filtration in situations when the measuring platform passed under: (**a**) road viaduct; (**b**) railway viaduct; where: *A*, *B*—acquired coordinates, A*, B*—their values after filtration, *l_b_*—base length, base slope, relative error of base length, and detector signal.

Data filtration was also performed for datasets with a partial loss of signal when the measuring platform passed under viaducts. The results of these measurements are shown in Figure 15. The straight track section selected for analysing was over 200 m long and the data collected when passing this section comprises nearly 600 samples. Due to signal loss and significant changes of coordinate values, each correct data detector **P**, based on SGF and windows with 5, 7, 9, and 11 points, precisely detected the range of incorrect measurement data. Figure 15 shows coordinates of receivers *A* and *B* composing the base vector after Whittaker filtration. Figure 15a represents the situation when the measuring platform passes under the road viaduct, while Figure 15b—under the lattice truss railway viaduct. Both figures present changes of the calculation base length l_b_* obtained from processed data A* and B*. For the measurements made when passing under the road viaduct, the maximum relative error of base length was 2.6%, while for the railway viaduct—1.1%.

To verify the GNSS results and their filtration, additional measurements of track axis coordinates were made using the spatial resection method and free total station. The total station was placed on selected points along the track, and the positions of these points were determined using the resection method for four points of Special Railway Surveying Grid. The prism was mounted on the track gauge meter, and relevant measuring offsets were taken into consideration. The measurement of prism coordinates was done every 10 m.

**Figure 16:**
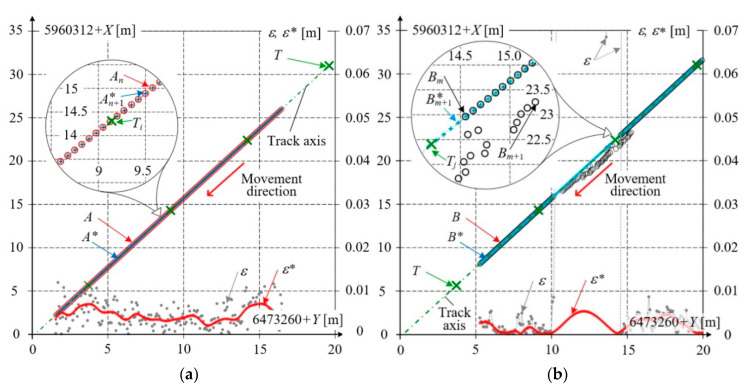
Measurement results obtained from receivers *A*, *B* and total station *T*, and distribution of absolute errors: *ε* for *A*, *B* and ε* for A*, B*: (**a**) receiver *A*; (**b**) receiver *B*.

Figure 16 shows the results of total station measurements, marked *T*. For the datasets from Figure 9 and Figure 14, collected on the straight horizontal track section, the track axis was determined from five successive measurements and described by the reference linear equation. Four successive measuring points and the track axis are marked in Figure 16. At the background of the track axis, the results of GNSS measurements for receivers *A* and *B* are shown, along with their values A* and B* obtained in the filtration process. The distance of the GNSS measurement point from the line defining the track axis, measured in the direction perpendicular to this axis, defines the absolute error *ε*.

Figure 16a shows the situation when all data recorded by receiver *A* are correct. The decrease of the relative error ε* for filtered values, as compared to the error *ε* for non–processed data can be seen. The value of error ε* reached a maximum of 7 mm. The track axes determined from data *A* and A* practically coincide with the reference axis calculated from total station data.

Figure 16b presents the results for receiver *B*. In the interval with incorrect data, the absolute error of non–processed data is large, up to 0.6 m. The absolute error ε* for filtered values only slightly exceeds 5 mm. The track axis determined from data *B* is shifted by a constant distance of 0.14 m with respect to the reference axis. For the data B*, processed and complemented in the manner resulting from the filtration algorithms, the track axis practically coincides with the reference axis.

The performed analysis testifies to the correctness of the adopted assumptions concerning detection and filtration algorithms applied to the acquired GNSS data.

## 5. Conclusions

New opportunities concerning mobile satellite measurements of railway tracks axes are provided by a measuring platform equipped with at least two GNSS receivers installed above bogie pivot pins, which define the base vector of the vehicle. This approach makes it possible to determine more precisely the track axis as well as the position and length of straight track sections, positions, lengths and radii of circular arcs, and positions, and types and lengths of transition curves. Moreover, the base vector can be used for qualitative evaluation of the obtained measurement results, i.e., identification of correct and incorrect data. Having greater numbers of receivers provides opportunities for better control of the results.

The sporadic appearance of samples with a relatively large deviations of coordinate values from those recorded by other receivers was observed. These errors were not caused by environmental disturbances. It has been shown that the sensitivity of the method is sufficient for both the identification of incorrect measurements and in the case of GNSS signal loss due to obstacles. The novel method developed to identify incorrect measurement results with the Savitzky–Golay filter is reliable, simple, easy to use and is characterised by high speed of numeric calculations (possible use of sparse matrices). The obtained incorrect data detector was used in the Whittaker smoothing algorithm. The performed analyses and the obtained results testify to the correctness of the method adopted for processing the data acquired by GNSS receivers.

Future work concerning mobile GNSS measurements can include implementation of automatic parametrization of the two developed filters. Also of some interest is the potential use of signal differentiation provided by the Savitzky–Golay filter for analysing smoothed signals with the Whittaker algorithm. It is also planned to use fusion of the filtered data with the results of measurements coming from independent inclinometers, accelerometers and/or INS modules to improve the quality of input data to then be used in algorithms of precise track axis evaluation. This also refers to corrections of longitudinal and lateral track inclinations resulting from GNSS receiver positions at a relatively large height above the track plane.

## Figures and Tables

**Table 1:** Position coordinates of GNSS receivers determined for stationary measurements.

Receiver	Coordinates *Y*, *X*		Standard Deviation	
	*Y* [m]	*X* [m]	*σ_Y_* [mm]	*σ_X_* [mm]
*A*	6,473,870.062	5,961,286.486	6.70	6.80
*B*	6,473,873.743	5,961,292.432	12.82	10.20
*C*	6,473,870.691	5,961,286.092	5.88	6.79
*D*	6,473,869.418	5,961,286.889	7.34	5.27
*E*	6,473,874.379	5,961,292.044	8.52	10.77
*F*	6,473,873.109	5,961,292.835	5.43	4.88

**Table 2:** Measured distances between receivers and their errors.

Section	Distance [mm]	GNNS Distance [mm]	Absolute Error Δ [mm]	Relative Error δ [%]
*AC*	750	742.2	7.8	1.04
*AD*		759.7	9.7	1.29
*BE*		745.0	5.0	0.67
*BF*		751.2	1.2	0.16
*CD*	1500	1501.9	1.9	0.03
*EF*		1496.2	3.8	0.25
*CE*	7000	7001.9	1.9	0.03
*AB*		6993.2	6.8	0.10
*DF*		6998.5	1.5	0.02
*BC*	7040	7036.4	3.6	0.05
*AE*		7048.7	8.7	0.12
*AF*		7042.3	2.3	0.03
*BD*		7030.7	9.3	0.13
*CF*	7159	7163.4	4.4	0.06
*DE*		7154.4	4.5	0.06
